# Impact of Cross-Linking of Collagen Matrices on Tissue Regeneration in a Rabbit Calvarial Bone Defect

**DOI:** 10.3390/ma14133740

**Published:** 2021-07-04

**Authors:** Masako Fujioka-Kobayashi, Elena Andrejova, Hiroki Katagiri, Benoit Schaller, Anton Sculean, Jean-Claude Imber, Niklaus P. Lang, Nikola Saulacic

**Affiliations:** 1 Department of Cranio-Maxillofacial Surgery, Inselspital, Bern University Hospital, University of Bern, 3012 Bern, Switzerland; masako.kobayashi@tky.ndu.ac.jp (M.F.-K.); elena.andrejova@students.unibe.ch (E.A.); katagiri@ngt.ndu.ac.jp (H.K.); benoit.schaller@insel.ch (B.S.); nplang@switzerland.net (N.P.L.); 2 Department of Oral and Maxillofacial Surgery, School of Life Dentistry at Tokyo, The Nippon Dental University, Tokyo 102-8275, Japan; 3 Advanced Research Center, School of Life Dentistry at Niigata, The Nippon Dental University, Niigata 951-8580, Japan; 4 Department of Periodontology, University of Bern, 3012 Bern, Switzerland; anton.sculean@zmk.unibe.ch (A.S.); jean-claude.imber@zmk.unibe.ch (J.-C.I.)

**Keywords:** collagen, cross-linking, tissue augmentation, bone formation

## Abstract

The cross-linking of collagen matrices (Cl_CM) may provide volume-stable enhanced defect regeneration when compared to non-cross-linked matrices (Ncl_CM). The aim of the present study was to investigate the bone forming potential of collagen matrices (CMs) and the effects of cross-linking CMs in a rabbit calvaria defect model. (1) Empty controls (*n* = 6), (2) Ncl_CM (*n* = 8), and (3) Cl_CM (*n* = 8) were selected to be observed for the healing in 10 mm critical-sized calvarial bone defects. The potential for the bone as well as the connective tissue formation were evaluated by micro-CT and histomorphometry at three months post-surgery. There were no statistically significant differences in terms of new bone volume in the defects between the groups. However, the Cl_CM induced significantly greater fibrous tissue regeneration (5.29 ± 1.57 mm^2^) when compared to the controls (3.51 ± 0.93 mm^2^) by histomorphometry. The remnants of collagen fibers with immune cells, including macrophages and giant cells, were occasionally observed in the Cl_CM group but not in the Ncl_CM group. In conclusion, the cross-linking of collagen did not influence the potential for bone formation. Nevertheless, Cl_CM might be advantageous for the maintenance of fibrous tissue volume without disturbing bone formation in the defects.

## 1. Introduction

Collagen matrices (CM) are routinely used as biomaterials to enhance wound healing and the regeneration of soft and hard tissue defects. In the dental field, a xenogeneic CM was recently developed for soft tissue augmentation and proved to be a suitable alternative to autogenous tissue transplants, maintaining tissue volume and thickness generally without adverse events such as infection [1,2,3,4,5]. Cross-linked CM (Cl_CM) has been developed for the improvement and lower shrinkage rate of non-cross-linked CM (Ncl_CM) [1,3]. The slower degradation of Cl_CM appeared to improve tissue volume stability for the long-term [1,6,7,8].

One of the disadvantages of the Cl_CM, however, is related to its lower biocompatibility when compared to Ncl_CM. For instance, it was reported that the glutaraldehyde cross-linked collagen membranes demonstrated lower cell attachment and viability when compared to non-cross-linked collagen membranes in human gingival fibroblasts [9]. Nevertheless, our previous in vitro study proved the excellent cytocompatibility of the chemically cross-linked Cl_CM as well as Ncl_CM on human THP-1-derived macrophages and gingival fibroblasts [10]. However, slight immune cell responses were observed with the higher mRNA levels of M1 markers, including interleukin (IL)-1 and IL-6, in the macrophages cultured on Cl_CM [10]. These data are in line with a recent publication reporting an early, slight inflammation after the implantation of bovine cross-linked collagen hemostats in Balb/c mice [11]. The biocompatibility of Cl_CM has also been documented in vivo, with obvious favorable soft and connective tissue integration and an adequate promotion of angiogenesis [1,3,12,13,14,15,16]. It was reported that soft tissue volume augmentation was obtained to a similar extent using Cl_CM and connective tissue graft (CTG) in preclinical and clinical studies [1,3]. For instance, Thoma et al. compared Cl_CM and CTG for their use in alveolar ridge augmentation around dental implants in dogs, which showed similar outcomes of soft tissue volume augmentation at the implant sites with minimal inflammatory reactions between two procedures for up to two months [1].

It is worth noting that collagen materials have been recognized to not only promote soft tissue regeneration but also support hard tissue regeneration, i.e., bone. Ncl_CMs are used for alveolar ridge preservation in order to maintain alveolar bone height and width after tooth extraction. It has recently been shown that a Cl_CM improved periodontal tissue regeneration when compared to an empty control in a two-wall intrabony defect model in dogs [17]. Additionally, it has been suggested that Cl_CM may be useful for soft tissue augmentation even in conjunction with guided bone regeneration (GBR) procedures [18]. As volume maintenance is essential for ridge preservation, a cross-linked volume-stable CM may serve this purpose better as opposed to non-cross-linked CMs. However, as Cl_CMs have primarily been developed for soft tissue augmentation, the effects of Cl_CM on bone formation in a critical-sized bone defect has not yet been investigated.

The aim of this study was to elucidate the bone forming potential of two collagen matrices in the form of sponges—one cross-linked (Cl_CM) and the other, non-cross-linked (Ncl_CM)—as compared to blood clot alone. Hence, these two types of CMs were tested in a critical-size bone defect at three months post-implantation by means of micro-CT and histological analysis, and were subsequently compared to an empty defect.

## 2. Materials and Methods

### 2.1. Materials

**Figure 1:**
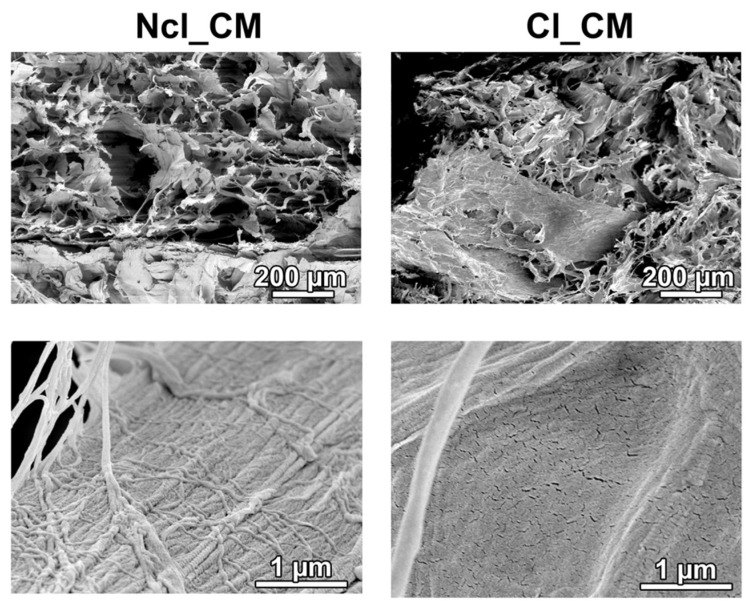
SEM images of the non-cross-linked collagen matrix (Ncl_CM) and cross-linked CM (Cl_CM) at low magnification (100×) and high magnification (30,000×).

Two compositions of CMs were kindly provided by Geistlich Pharma AG, Wolhusen, Switzerland. Both consisted of collagen type I and III; one of them was non-cross-linked (Ncl_CM) and the other was chemically cross-linked (Cl_CM). Both CMs were porous, resorbable biomaterials, specifically designed for soft-tissue augmentation. The CMs were morphologically observed via scanning electron microscopy (SEM) (DSM 982, Zeiss, Oberkochen, Germany) (Figure 1). Both Ncl_CM and Cl_CM showed three-dimensional (3D) mesh patterns at low magnification, whereas Cl_CM further included relatively dense structures of collagen fibers at high magnification.

### 2.2. Animals

Twelve New Zealand White female rabbits, approximately 16 weeks of age (2.6–3.4 kg), were used in the present study. The sample size of animals (sham: 8 rabbits, Ncl_CM: 8 rabbits, and Cl_CM: 8 rabbits) were estimated by using G*power [19]. Unfortunately, due to the unexpected death of an animal after surgery, the total sample number of 22 was used for the evaluation instead.

The present study was approved by the Committee for Animal Research, Canton of Bern, Switzerland (Nr: BE 89/17). The NC3Rs, UK guidelines, and ARRIVE guidelines for preclinical in vivo studies were considered. After the acclimatization period, the animals were housed without excessive or disturbing noises, fed a standard diet, and given water ad libitum.

### 2.3. Anesthesia

Anesthesia was performed, as previously reported [20]. Briefly, the animals were premedicated subcutaneously (s.c.) with methadone (0.3 mg/kg) and dexmedetomidine (100 g/kg) mixed with ketamine (15 mg/kg). General anesthesia was maintained with isoflurane vaporized in pure oxygen through a Jackson Rees modified T-piece breathing system, targeting a maximal Et Iso of 1–1.3%. Ropivacaine 0.75% was locally administered on the surgical site.

### 2.4. Surgical Procedures

**Figure 2:**
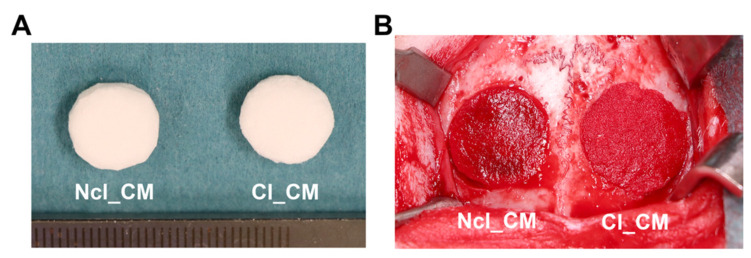
(**A**) Pre-shaped Ncl_CM (**left**) and Cl_CM (**right**) before the implantation (φ = 10 mm, thickness = 3 mm). **(B**) Image of the implanted CMs during surgery.

The skin of the head was incised from the nasal bone to the mid-sagittal crest, and the parietal bone was exposed after the periosteum elevation. Two critical-sized (10 mm diameter) bone defects were prepared in the parietal bones with a trephine drill, with maximal care not to injure the dura mater. Both CM biomaterials were pre-shaped with a scalpel into 10 mm diameter and 3 mm thickness cylinder (Figure 2A), and implanted in the defects without excessive pressure (Figure 2B). The applied treatment modalities, (1) empty control (*n* = 6), (2) Ncl_CM (*n* = 8), and (3) Cl_CM (*n* = 8), were randomly allocated. The wound was sutured in two layers using 4–0 Vicryl^®^ (Ethicon, Somerville, NJ, USA) and 4–0 Monocryl^®^ sutures (Ethicon). Furthermore, the surfaces of the wound were covered with a spray film dressing (OPSITE^®^ SPRAY, Smith & Nephew, London, UK).

### 2.5. Postoperative Procedures

Perioperatively, antimicrobial prophylaxis (procaine penicillin 150,000 IU/mL + benzathine penicillin 150,000 IU/mL; 0.01 mL/kg s.c, Duplocillin^®^, MSD Animal Health, Luzern, Switzerland) were applied. As postoperative analgesia, meloxicam (Metacam^®^, Boehringer Ingelheim, Ingelheim, Germany) 0.5 mg/kg s.c., once daily during postoperative period of 4 days, and buprenorphine (Temgesic^®^, Rechitt Benckiser, Wallisellen, Switzerland) 20 g/kg s.c., every 8 h during postoperative period of 3 days were administered. The animals were euthanized at 3 months post-surgery with an overdose of pentobarbital 120 mg/kg i.v., after the premedication with ketamine 65 mg/kg and xylazine 4 mg/kg s.c.

### 2.6. Micro-CT Analysis

The collected calvarial samples were fixed in a 10% neutral formalin solution for 7 days at room temperature and kept in 70% ethanol at 4 °C. The micro-CT scans were performed by utilizing a desktop cone beam scanner (micro-CT 40, ScancoMedical AG, Brüttisellen, Switzerland). The micro-CT DICOM images (voxel size; 18 μm) were then analyzed and 3D reconstructed by a software (Amira ver. 2019, Thermo Fischer Scientific, Waltham, MA, USA). The volume of interest (VOI) was set as 10 mm diameter, full thickness cylinders, selected corresponding to the dimensions of the bone defect sites. Two-dimensional parameters included the defect closure measured on the horizontal plane (DC, relative % to whole defect length; 10 mm) and bone height (BH) measured on the sagittal plane, in the middle 5 mm (BH middle) and the lateral part of the defect (BH lateral). Three-dimensional parameters including bone volume (BV, mm^3^), BV fraction (BV/TV, ratio of the segmented BV to the total tissue volume), and bone density (BD, mgHA/ccm at the defect site), were calculated.

### 2.7. Histological Processing and Histomorphometric Analysis

**Figure 3:**
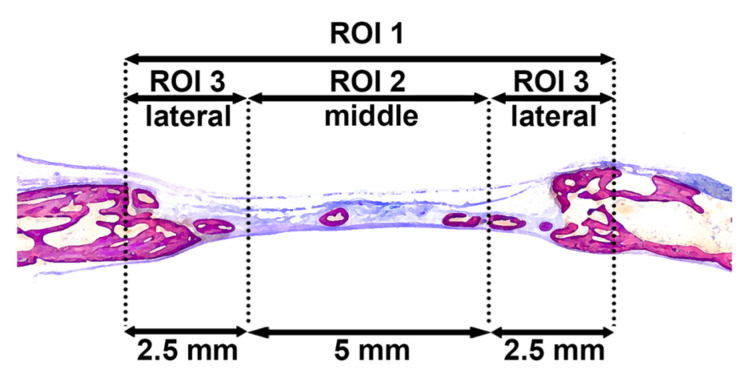
ROIs for histomorphometry. ROI 1 represents the whole defect area (10 mm), ROI 2 is middle area of the defect (5 mm), and ROI 3 is lateral area of the defect (2.5 mm + 2.5 mm).

All calvarial samples were dehydrated in ascending concentrations of ethanol, and embedded in methyl methacrylate (MMA) without decalcification. For accurate bone histomorphometry, only the undecalcified sections were prepared in the present study. The MMA-embedded blocks were cut sagittally into 1000 μm thick ground sections in the middle of the defects using a diamond saw (VC-50; LECO, St. Joseph, MI, USA). After mounting on acrylic glass slabs, the sections were ground and polished to a final thickness of 200 μm, then stained with toluidine blue and fuchsin. The images were captured under a digital microscope (VHX-6000, Keyence, Japan). Histomorphometry was performed by Photoshop CC software (Adobe, San Jose, CA, USA) in the region of interest (ROI 1) corresponding to 10 mm initial defect sites, as previously reported [20,21] (Figure 3). Linear parameters included horizontal defect closure (HDC, %), new bone height in the middle 5 mm area (ROI 2; NBH middle, mm), NBH in the lateral 5 mm area (ROI 3; NBH lateral, mm), maximal connective tissue height (maximal CTH, mm), maximal adipose tissue height (maximal ATH, mm), and maximal fibrous tissue height (maximal FTH, mm) were determined. Furthermore, as area parameters, new bone area (NBA, mm^2^), bone marrow area (BMA, mm^2^), residual material area (RMA, mm^2^), connective tissue area (CTA, mm^2^), adipose tissue area (ATA, mm^2^), and fibrous tissue area (FTA, mm^2^) were calculated in ROIs.

### 2.8. Statistical Analysis

The floating bars in the graphs represent the minimum and maximum values with the mean values for all quantitative data. The statistical analysis was performed using one-way analysis of variance (ANOVA) with Tukey test by a statistical software (GraphPad Prism 8.0; GraphPad Software, Inc., La Jolla, CA, USA). The *p* values < 0.05 were considered significant.

## 3. Results

### 3.1. General Observation during and Post-Surgery

During surgeries, dried Ncl_CM and Cl_CM placed into the defect were readily and completely soaked with the surrounding blood. Both materials as scaffolds of stable blood clot showed slight swelling in the defects; however, the periosteum and skin could be easily closed without major tension. In general, wound healing was uncomplicated, without signs of inflammation, infection, wound dehiscence or exposure of the surgical sites post-operatively.

### 3.2. Micro-CT Analysis

**Figure 4:**
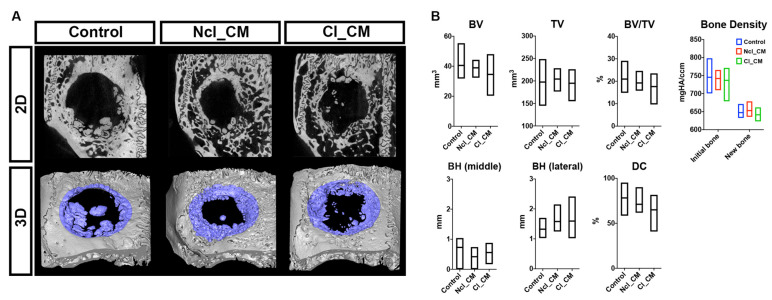
The micro-CT analysis. (**A**) Representative images of the micro-CT data in each group; the 2D plane (upper row) and 3D-reconstructed views (lower row; the blue color shows new bone in VOI). (**B**) The quantified data of the micro-CT analysis. Bone volume (BV, mm^3^), total volume (TV, mm^3^), BV/TV (%), bone density (mmHA/ccm), bone height (BH, mm) in middle area, BH in lateral area, and defect closure (DC, %) were calculated in each group at three months post-surgery. No significant differences were observed in any parameters among the tested groups.

The specimens were first analyzed by micro-CT (Figure 4). All groups showed some new bone formation in the bone defects. However, none of the defects led to complete closure after three months. The control empty samples tended to induce smooth new bone islands in the defects, while Ncl_CM and Cl_CM samples demonstrated relatively rough new bone surfaces where the materials were implanted, usually connected to the initial peripheral bone edges (Figure 4A). There were no statistically significant differences in any quantifiable parameter among the three tested groups (Figure 4B). BV/TV in the control group, for example, demonstrated 20.96 ± 5.47%, while it was 19.23 ± 3.08% in Ncl_CM and 17.63 ± 4.46% in Cl_CM. The new bone tissue of the empty control group tended to grow toward the middle of the defect region, but the lateral area of bone looked relatively thin when compared to the defects filled with CMs. Both Ncl_CM and Cl_CM showed relatively thick new bone in the lateral area of the defects. However, new bone in middle area of the defects was rarely observed in both Ncl_CM and Cl_CM groups. The Bone Density values revealed that the new bone at three months post-surgery was still immature (low mineral density) when compared to the reference bone (Figure 4B).

### 3.3. Histological Analysis

**Figure 5:**
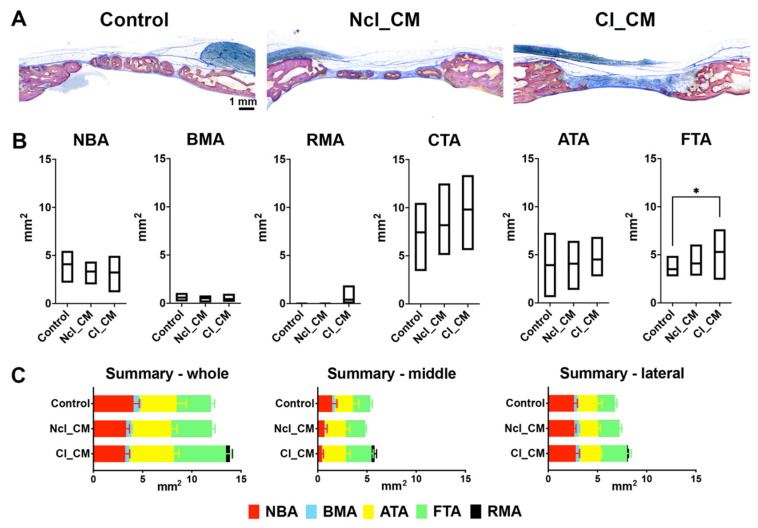
(**A**) Overviews of toluidine blue and fuchsin staining of the defect on the sagittal plane at three months post-surgery. (**B**) Histomorphometry of area parameters including new bone area (NBA, mm^2^), bone marrow area (BMA, mm^2^), residual material area (RMA, mm^2^), connective tissue area (CTA, mm^2^), adipose tissue area (ATA, mm^2^), and fibrous tissue area (FTA, mm^2^). * denotes significant difference between the groups; *p* < 0.05. (**C**) Summary of area parameters in whole defects (ROI 1), middle area of the defects (ROI 2), and lateral area of the defects (ROI 3).

**Figure 6:**
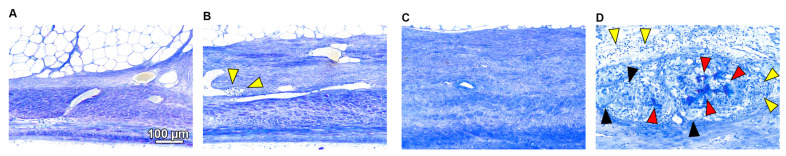
The magnified views of toluidine blue and fuchsin staining in the middle area at high magnification (500×) in (**A**) Control (empty), (**B**) Ncl_CM, and (**C**,**D**) Cl_CM groups at three months post-surgery. The yellow arrowheads show lymphocytes, red arrowheads indicate residual materials, and black arrowheads show multinucleated giant cells (MNGCs).

**Figure 7:**
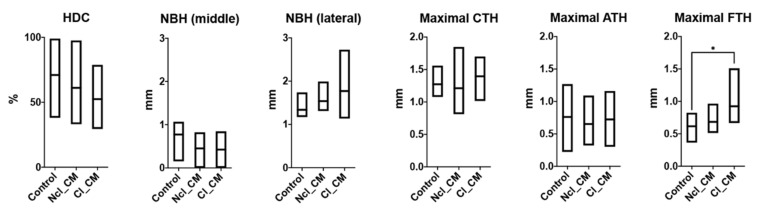
Histomorphometry of linear parameters including horizontal defect closure (HDC, %), new bone height in the middle area (NBH middle, mm), NBH in the lateral area (NBH lateral, mm), maximal connective tissue height (Maximal CTH, mm), maximal adipose tissue height (Maximal ATH, mm), and maximal fibrous tissue height (Maximal FTH, mm) at three months post-surgery. * denotes significant difference between the groups; *p* < 0.05.

Similar results were observed in the histological analysis as compared to the micro-CT analysis (Figure 5, Figure 6 and Figure 7). The new bone stretched out to the middle of the defects in the empty controls, whereas Cl_CM maintained thicker new bone in the peripheral area (Figure 5). Some residual materials remained, especially in the middle area in the Cl_CM group (Figure 5). No obvious residual materials were found in the Ncl_CM group. There were no statistically significant differences in NBA, BMA, RMA, CTA, and ATA (Figure 5B). However, a trend could be observed towards increased CTA and ATA at the expense of NBA and BMA in the case of the CMs, with Cl_CM being more pronounced. Statistically significantly higher FTA was found in Cl_CM (5.29 ± 1.57 mm^2^) when compared to the empty controls (3.51 ± 0.93 mm^2^, * *p* = 0.0337) (Figure 5B).

Similarly, in the middle of the defects, thicker fibrous tissue was observed in Cl_CM (0.92 ± 0.28 mm) when compared with both empty controls (0.62 ± 0.18 mm, * *p* = 0.0367) and Ncl_CM (0.68 ± 0.15 mm, *p* = 0.0849) (Figure 6 and Figure 7). No residual materials were observed for any of the Ncl_CM specimens. Lymphocytes were, however, occasionally detected in the connective tissue in the Ncl_CM group (Figure 6B). The remnants of Cl_CM were encapsulated in the connective tissue and accompanied by numerous lymphocytes and multinucleated giant cells (MNGCs) (Figure 6D). No statistically significant differences were observed in the linear parameters except for maximal FTH between the groups (Figure 7).

## 4. Discussion

The present experimental study evaluated in a critical-sized defect model, the bone forming potential of two collagen matrices (Cl_CM; Ncl_CM) as compared with empty defects, and investigated the potential positive effects of the increased volume stability obtained through the chemical crosslinking of a CM. No statistically significant difference could be detected in terms of new bone formative potential between the two CMs and the empty defect in the whole defect. However, the obvious trend was that in the case of both collagen scaffold groups, less bone was formed especially in the middle area of the defect when compared to the empty defect group at three months after augmentation. This effect was more pronounced in the case of the cross-linked collagen (Cl_CM) when compared with the non-cross-linked collagen (Ncl_CM). Taken together, these findings indicate a delayed bone formation in the presence of CMs in the middle portion of the defect.

The Cl_CMs were previously introduced for soft tissue augmentation, as an alternative to autogenous soft tissue grafts, while the studies performed showed that the matrices may also provide space and preserve the tissue volume [1,3]. As volume maintenance is also essential for alveolar ridge preservation, it was hypothesized that Cl_CM may serve the same purpose when placed into bony defects. Unfortunately, the overall bone formation was unaffected by the scaffolding with the cross-linking of CMs, if not even negatively impacted. Moreover, the analysis revealed that this negative trend was less evident in the case of Ncl_CM. Given that (i) Cl_CM is chemically cross-linked, (ii) chemical cross-linking is known to modulate the collagen binding region for α2β1 integrin [22], the latter functioning as primary platelet adhesion receptor [23], we assume less efficient blood clot formation as the potential cause for the observed trend, considering blood clot formation as the essential first step in bone formation.

Despite the observed delay in overall bone formation kinetics, it has to be emphasized that Cl_CM allowed for a horizontal and vertical ingrowth of new bone at the defect borders in contrast to the Ncl_CM and empty defects. This indicates that a non-cross-linked highly porous CM, despite occupying the whole defect in the very early phase of bone healing, resorbs too early and cannot prevent the collapse of connective tissue into the defect site. In combination with a missing barrier, this leads to an outcome similar to an empty defect. A stable, highly porous collagen matrix may prevent the collapse of the surrounding tissue into the defect area. Clearly, the resorption kinetics of the tested Cl_CM in this study needs to be balanced to the bone formation, continuously maintaining the space for new bone. A functional barrier excluding fibrous tissue seems to be mandatory for successful bone augmentation, as significantly more fibrous tissue in the defect has been found when compared to Ncl_CM as well as empty defects.

The in vitro assay was not chosen in the present study to compare the Ncl_CM and Cl_CM for osteogenic potential. The effect of the different Ncl_CM and Cl_CMs on cell behavior in macrophages and gingival fibroblasts were previously investigated [10]. It was unfortunately revealed that the Ncl_CMs are resorbed in cell culture media within a couple of days, and this quick resorption process influenced cell viability. Therefore, an in vitro osteoblast differentiation assay was not performed as it requires long-term culture periods. However, it is interesting that the osteogenetic potential was reportedly promoted by bone marrow-derived multipotent stromal cells cultured on cross-linked collagen membranes in comparison to non-cross-linked collagen membranes, supported by higher alkaline phosphatase (ALP) expression, calcium deposition, and angiogenic potential [24]. A 10 mm critical-size defect in rabbit calvariae is one of the best established models to evaluate the bone regeneration potential of biomaterials [25] and was chosen for this study. The bone defect models of smaller animals could not be considered to evaluate the bone formation potential of the tested CMs because of the thickness of the materials, which are not membranes. There was, however, no obvious benefit of CM application, cross-linked or non-cross-linked, on the formation of new bone in the present in vivo study, as indicated by both micro-CT analysis and histomorphometry. Nevertheless, in our study, we did not use barrier membranes to exclude the ingrowth of soft tissue, as requested conceptually by the guided bone regeneration (GBR) approach. Thus, despite the absence of collagen barrier membranes, the used CMs performed as volume-stable scaffolds that occupied the entire bone defects. On the other hand, both CMs did not hinder the formation of new bone as compared to the empty controls. The present study, therefore, does not exclude the fact that the CMs may provide a positive impact on bone formation in less demanding defect models.

Despite the fact that the tested CMs did not influence bone formation, the present results supported findings from previous studies on soft tissue regeneration—both CMs were incorporated into the newly formed tissue without distinctive borders to the CM or encapsulation [1,26]. Interestingly, the Cl_CMs yielded the highest CTA, ATA, and FTA among the modalities tested. In contrast to Ncl_CM, the difference of the results of Cl_CM reached statistical significance for both FTA and FTH when compared to the empty control. Thus, modification of the degradation kinetics of CMs by chemical cross-linking and its influence on the formation of soft tissue has been confirmed. These findings further point to the necessity to exclude soft tissue ingrowth, in line with the guided bone regeneration concept.

In the present study, MNGCs and a mononuclear infiltrate were detected in the Cl_CM samples around the remnants of the materials in the middle defect area. The mononuclear infiltration around collagen fiber remnants suggests an extended inflammation, the latter potentially driven by M1 macrophages causing a delay in tissue integration. In that respect, our data are in agreement with results reported for cross-linked collagen membranes [27]; the higher degree of cross-linking was found responsible for the prolonged periods of resorption of the remnants [28], stimulating in parallel pro-inflammatory reactions and the activation of macrophage colonies more than their non-cross-linked counterparts [29]. These findings have been confirmed in the in vivo analysis of bovine cross-linked collagen sponges, which caused a benign and transient immune reaction [11]. In contrast, non-cross-linked collagen membranes were completely degraded by collagenases exerting only a small inflammatory reaction, without a foreign body reaction [29]. The same complete resorption of Ncl_CM was also observed in the present study. In conclusion, the cross-linked as well as non-cross-linked collagen matrices display distinct biological behavior, corroborating earlier findings on the respective collagen membranes.

When implanted into the soft tissues, MNGCs expressing CD86, cathepsin K, and tartrate-resistant acid phosphatase (TRAP) were found to be responsible for the degradation of CMs [15]. Macrophages are known to regulate the vascularization [30] and the rapid synthesis of collagen fibrils [31]. Furthermore, neutrophils also contribute to inflammation, macrophage recruitment, M2 macrophage polarization, inflammation resolution, and angiogenesis during tissue regeneration following the implantation of the biomaterials in the body [32]. Ye et al. observed differences in the infiltration of neutrophils between two dermal sheep collagen membranes cross-linked by glutaraldehyde and hexamethylene diisocyanate in murine subcutaneous tissue [33]. It was demonstrated that neutrophils upregulated the expression of interferon (IFN)-γ, which activates macrophages [33]. However, comprehensive knowledge is lacking about the subsequent healing events, and the immuno-cells behavior in the pores of the CMs is not yet fully understood. An appropriate balance between the resorption speed of biomaterials and the degree of bone formation/replacement might be beneficial for bone regeneration, depending on each clinical situation [34]. The present study did not clarify the functions of the related immune cells and bone forming cells because of the limited observation periods and assay procedures. Further studies are therefore urged to fully understand the immuno-function of CM on tissue regeneration.

## 5. Conclusions

The present findings revealed that both collagen matrices, Ncl_CM and Cl_CM, did not improve bone formation when compared to the sham control group for three months. However, the Cl_CM showed slow resorption promoting connective tissue augmentation, without significantly disturbing bone formation in critical-size calvaria defects in rabbits.

## Data Availability

Data sharing not applicable.
